# Emerging trends and research foci of oncolytic virotherapy for central nervous system tumors: A bibliometric study

**DOI:** 10.3389/fimmu.2022.975695

**Published:** 2022-09-06

**Authors:** Kunming Cheng, Huan Zhang, Qiang Guo, Pengfei Zhai, Yan Zhou, Weiguang Yang, Yulin Wang, Yanqiu Lu, Zefeng Shen, Haiyang Wu

**Affiliations:** ^1^ Department of Intensive Care Unit, The Second Affiliated Hospital of Zhengzhou University, Zhengzhou, China; ^2^ Department of Neurosurgery, Affiliated Hospital 2 of Nantong University and First People’s Hospital of Nantong City, Nantong, China; ^3^ Department of Orhopaedic Surgery, Baodi Clinical College of Tianjin Medical University, Tianjin, China; ^4^ Department of Clinical College of Neurology, Neurosurgery and Neurorehabilitation, Tianjin Medical University, Tianjin, China; ^5^ Department of NeuroSpine Surgery, Tianjin Huanhu Hospital, Tianjin, China; ^6^ Department of Graduate School, Tianjin Medical University, Tianjin, China; ^7^ Department of Neurosurgery, Tianjin Huanhu Hospital, Tianjin, China; ^8^ Department of Graduate School, Sun Yat-sen University, Sun Yat-Sen Memorial Hospital, Guangzhou, China

**Keywords:** oncolytic virus, central nervous system, tumors, immunotherapy, bibliometric analysis, hotspots

## Abstract

**Background:**

Central nervous system tumor (CNST) is one of the most complicated and lethal forms of human tumors with very limited treatment options. In recent years, growing evidence indicates that oncolytic virotherapy (OVT) has emerged as a promising therapeutic strategy for CNSTs. And a considerable amount of literature on OVT-CNSTs has been published. However, there are still no studies summarizing the global research trends and hotspots of this field through a bibliometric approach. To fulfill this knowledge gap, bibliometric analysis was conducted based on all publications relating to OVT-CNSTs since 2000s.

**Methods:**

We searched the Web of Science Core Collection for all relevant studies published between 2000 and 2022. Four different tools (online analysis platform, R-bibliometrix, CiteSpace and VOSviewer) were used to perform bibliometric analysis and network visualization, including annual publication output, active journals, contribution of countries, institutions, and authors, references, as well as keywords.

**Results:**

A total of 473 articles and reviews were included. The annual number of publications on OVT-CNSTs showed a significant increasing trend. *Molecular Therapy* and *Cancer Research* were the most active and co-cited journals, respectively. In terms of contributions, there is no doubt that the United States occupied a leading position with the most publications (n=307, 64.9%) and the highest H-index (57). The institution and author that contributed the largest number of publications were Ohio State University and Chiocca EA, respectively. As can be seen from citation analysis, the current studies mainly focused on preclinical and phase I/II clinical results of various oncolytic virus for CNSTs treatment. Keywords co-occurrence and burst analysis revealed that the following research topics including immunotherapy, T-cells, tumor microenvironment, vaccine, blood-brain-barrier, checkpoint inhibitors, macrophage, stem cell, and recurrent glioblastoma have been research frontiers of this field and also have great potential to continue to be research hotspots in the future.

**Conclusion:**

There has been increasing attention on oncolytic viruses for use as CNSTs therapeutics. Oncolytic immunotherapy is a topic of great concern in this field. This bibliometric study provides a comprehensive analysis of the knowledge base, research hotspots, development perspective in the field of OVT-CNSTs, which could become an essential reference for scholars in this area.

## 1 Introduction

Central nervous system tumor (CNST) is one of the most lethal tumors across the globe with very limited therapeutic options. According to statistics, CNSTs account for 1.35% of human malignancies and 2.95% of cancer-related death (1). Among various types of CNSTs, glioma is the most common and fatal primary brain tumor in adults, responsible for more than 88% of brain tumor-related deaths (2). In general, despite aggressive therapy, gliomas are rarely curable especially for glioblastoma (WHO grade IV). The unsatisfying clinical therapeutic effect may have been due to several reasons such as strong invasiveness, easy relapse owing to the self-renewal and multi-lineage differentiation properties of glioma stem cells (GSCs), low drug delivery potency restricted by the blood-brain barrier (BBB), as well as the “immune privileged” state of the brain (3–6). Although considerable advances in the treatment of CNSTs have been achieved over the past decades, the prognosis of CNSTs patients remains very poor, highlighting the urgency to explore novel strategies in anti-tumor therapy (7, 8).

One promising strategy is to utilize viruses as oncolytic agents for the treatment of CNSTs. Oncolytic virotherapy (OVT) exploits the oncolytic properties of certain viruses including naturally occurring or genetically engineered viruses to selectively infect, replicate in, and kill cancer cells while sparing normal cells (9, 10). In addition, during the process of inducing tumor cell lysis, OVT also promotes the release of tumor-related antigens in the tumor microenvironment, which in turn activates the body’s immune system to initiate the anti-tumor immune response. Collectively, oncolytic viruses can exert their tumor-specific killing activities through a direct mechanism of cell lysis and indirect mechanisms mediated by the host immune system (11–13). In the recent 20 years, research on engineering oncolytic virus has achieved significant breakthroughs. In 2015, The US Food and Drug Administration (FDA) and European Medicines Agency (EMA) approved the first oncolytic virus, talimogine laherparepvec (T-VEC), an engineered immunostimulatory oncolytic type I herpes simplex virus, for the treatment of malignancies, which has underscored the therapeutic potential and safety of OVT for fighting cancer (14). In the field of neuro-oncology, the anticancer properties of oncolytic virus have also attracted a great deal of interest. To our knowledge, diverse oncolytic viruses have been introduced in clinical trials for progressive or recurrent CNSTs (15, 16). More encouragingly, a single-arm phase II clinical trial that evaluated the safety and efficacy of herpes simplex virus-1 (HSV-1, G47Δ) in patients with residual or recurrent glioblastoma has been completed in Japan recently (17). Results of this clinical trial showed that the 1-year-survival rate of these 13 subjects exceeded 90% without obvious side effects. Subsequently, in June 2021, G47Δ was officially approved by the Japan’s Ministry of Health, Labour and Welfare (MHLW) for the treatment of malignant glioma, which was the world’s first oncolytic agent approved for CNSTs.

In view of this, OVT in CNSTs (hereafter OVT-CNSTs) has received growing attention and a large number of related studies have been published in recent years. Nevertheless, in the face of massive information, researchers require a significant amount of time to read the academic documents to keep abreast of the latest developments in their fields. Although several scholars have adopted meta-analysis or systematic reviews to discuss the research progress on OVT-CNSTs, the profundity of such summaries is relatively limited as many researchers’ experiences are generally restricted to a particular direction, lacking the holistic understanding across the entire domain (18). Meanwhile, systematic reviews are also not able to provide some important information such as the main contributors including authors, institutions and countries, the dynamic development process, as well as the research hotspots and frontiers of a certain field (4). It is worth noting that, bibliometric analysis, as a scientific evaluation method, is able to compensate the deficiency.

Bibliometrics, first defined by Pritchard in 1969, is a comprehensive analytical method that integrates mathematics, philology and statistics (19–21). This method is capable of quantifying the distribution and characteristics of scientific information from multiple perspectives, and then reveals the knowledge structure, evolving trends, and potential hotspots of a certain field (22–24). In recent years, as more and more open-source bibliometric software becoming available, bibliometrics has been frequently applied in biomedical fields (25, 26). Take neuro-oncology as an example, Burak Atci et al. have conducted a bibliometric analysis of the top 50 most-cited articles on low-grade glioma from 1992 to 2013 (27). While Feng and colleagues have performed a more comprehensive analysis based on all glioma-related publications from 1994 to 2018. Their results demonstrated that studies on glioma have expanded rapidly over the past 25 years (28). In addition, there are also several bibliometric studies investigating the global research trends on therapeutic interventions of brain tumors such as endoscopic endonasal surgery (29), gamma knife (30), and intra-arterial drug delivery (31). However, to our knowledge, no prior studies have yet been performed to analyze the field of OVT-CNSTs through bibliometric approach.

**Figure 1:**
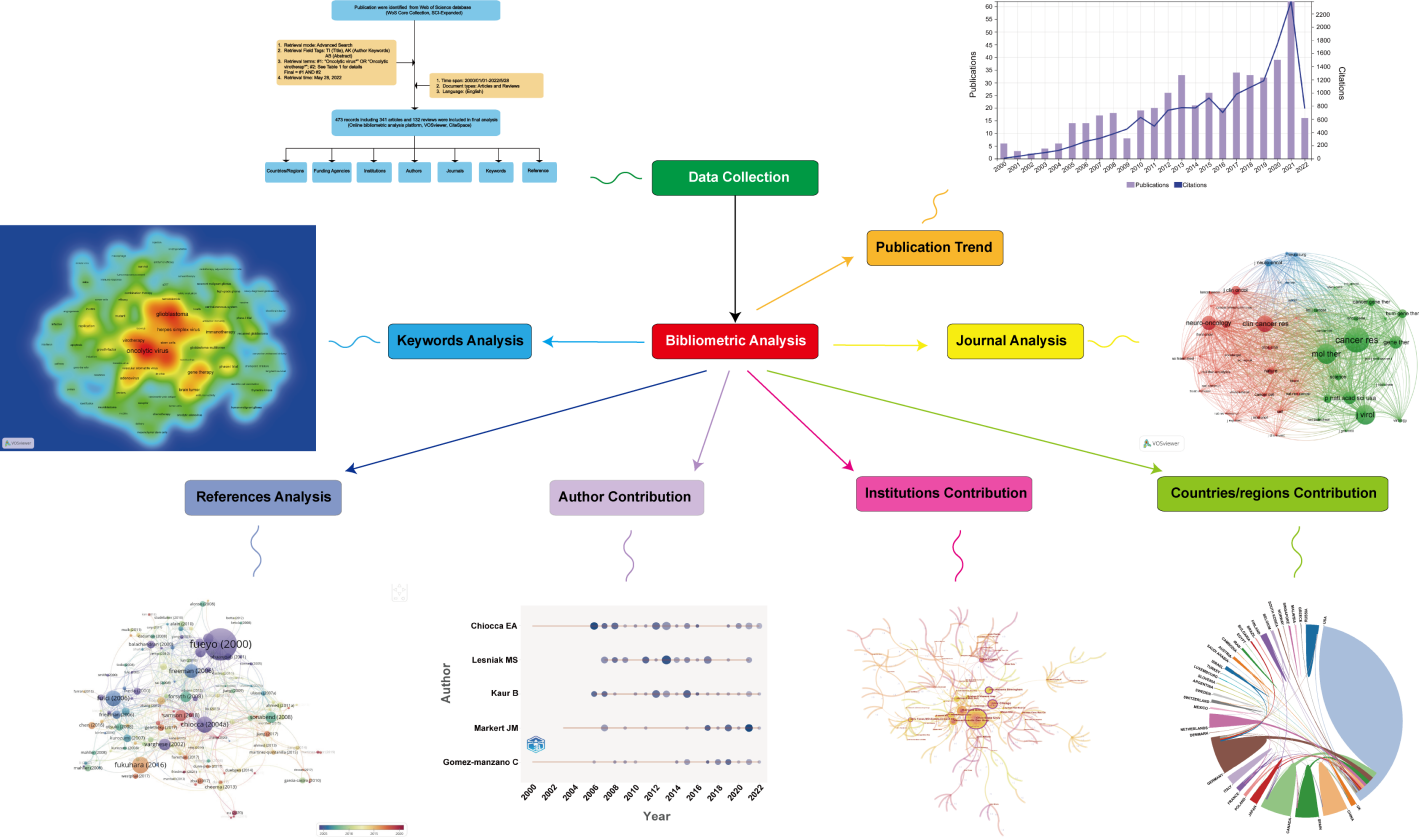
Graphic abstract of current study.

Thus, to fulfill this knowledge gap, we decided to conduct a bibliometric analysis based on literature regarding OVT-CNSTs from 2000 to 2022. Graphic abstract for the present study was shown in **Figure 1**. In brief, the main aim of the present study is to answer the following questions.

(1) How about the global research dynamics in the field of OVT-CNSTs?
(2) Who are the most popular and influential journals in this domain?
(3) Who are the main contributors from the level of countries, institutions or authors?
(4) What are the main research directions and focuses currently?
(5) What are the most concerned research frontiers and hotspots in the future?

## 2 Materials and methods

### 2.1 Data source and searching strategies

**Figure 2:**
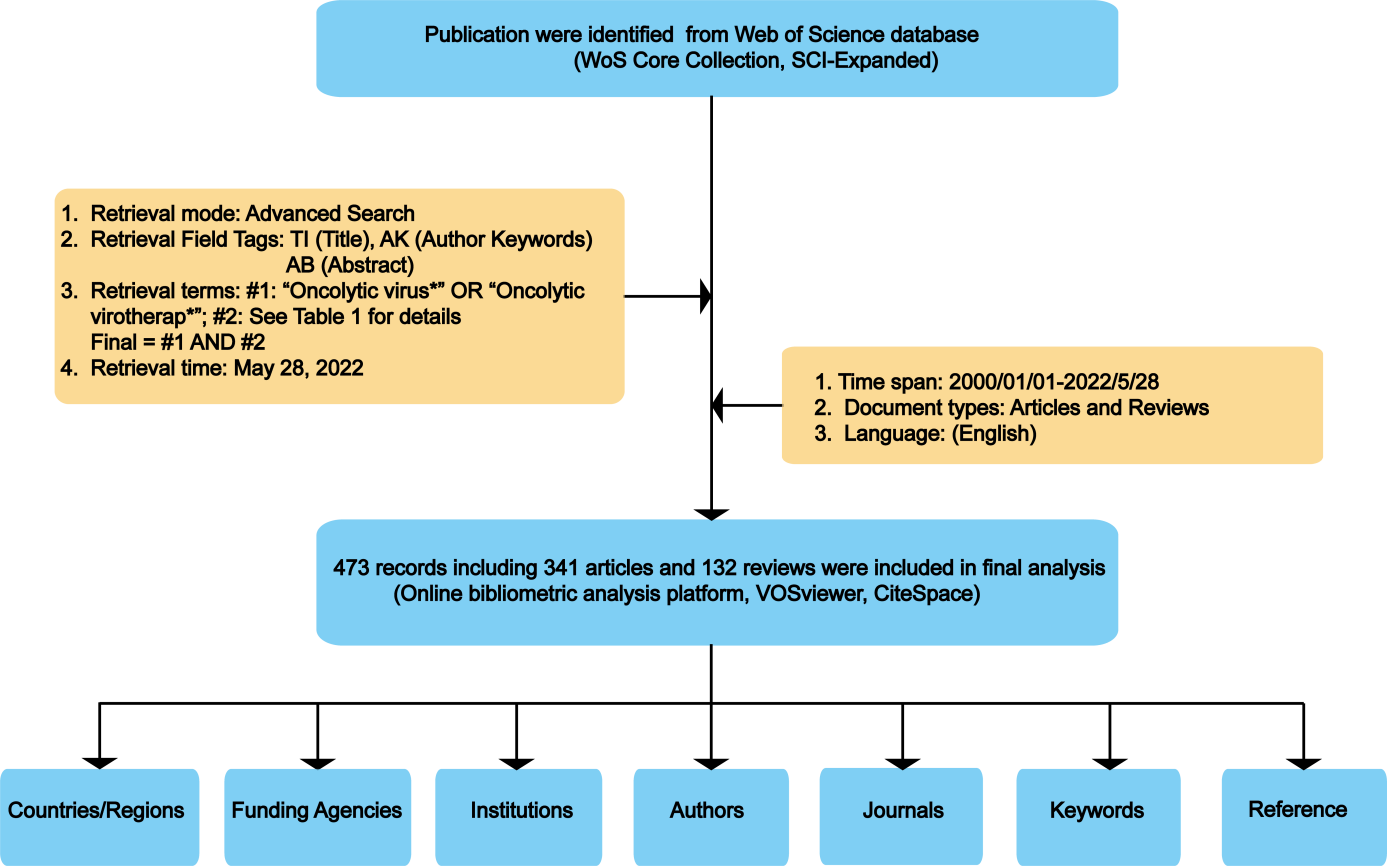
Detailed process of literature enrollment and data screening.

Among databases that could meet the requirements of global-level analyses, previous studies have indicated that Web of Science Core Collection (WoSCC, Clarivate Analytics) was the most appropriate one for bibliometric analysis (32, 33). Therefore, in the present study, all publications regarding OVT-CNSTs were retrieved from the Science Citation Index Expanded of WoSCC. In order to avoid biases caused by daily updates in the database, all retrieval operations and data downloads were completed within one day (May 28, 2022). Publications related to OVT-CNSTs were collected based on the titles (TI), abstract (AB) and author keywords (AK) with the several search terms and wildcard character such as “*” and “NEAR”. Search terms were determined through reviewing relevant literature and discussing with experts in OVT-CNSTs. Detailed search terms were shown in **Table 1**. The search period was restricted from 2000 to 2022. Language was limited to English and the article type was restricted to article or review (other article types including case reports, news items, book chapters, corrections, etc., were excluded). Duplicate documents were also identified and deleted. The detailed process of literature enrollment and data screening was illustrated in **Figure 2**.

**Table 1:** Search strategy and detailed search terms.

Strategy	Terms
**A**	“Oncolytic virus*” OR “Oncolytic virotherap*”
**B**	((brain OR “central nervous system” OR CNS OR intracranial) NEAR/1 (cancer* OR anticancer* OR tumor* OR tumour* OR oncology OR neoplasm* OR carcinoma* OR lymphoma*)) OR meningioma* OR astrocytoma* OR oligodendroglioma* OR oligoastrocytoma* OR glioma* OR glioblastoma* OR neuroblastoma* OR “leptomeningeal disease*” OR “leptomeningeal carcinomatosis*” OR “ependymoma*” OR “subependymoma*” OR “gangliocytoma*” OR “ganglioglioma*” OR “ganglioneuroma*” OR “ganglioneuroblastoma*” OR “chordoma*” OR “notochordal cell tumor*” OR “notochordal cell tumour*” OR “schwannoma*” OR “neurilemmoma*” OR “neurinoma*” OR “spinal cord tumor*” OR “spinal cord tumour*” OR “extradural tumor*” OR “extradural tumour*” OR “intradural extramedullary tumour*” OR “intradural extramedullary tumor*” OR “intradural intramedullary tumour*” OR “intradural intramedullary tumor*”
**C**	A AND B

Here, we consider central nervous system tumors in a broader sense and cover all tumor types in the central nervous system including meningiomas, gliomas, and spinal cord tumors. The wildcard character (*) that could be substituted for any other characters and allows variable endings of keywords was used. For example, “glioma*” would also return the terms of “glioma” and “gliomas”. The wildcard character of NEAR/1 was used to search for two words, in an arbitrary order, separated by a maximum of one term (e.g., CNS NEAR/1 tumor* would have identified “CNS tumor*” and “CNS malignant tumor*”).

### 2.2 Data extraction and collection

Literature screening, data filtering, and quality evaluation were carried out independently by two researchers. Bibliometric data including date of publication, annual number of publications/citations, countries/regions, institutions, authors, journals, funding agencies, keywords, references and research areas were obtained from these documents. Several inherent limitations of WoSCC were resolved manually. For instance, papers originating from Hong Kong, Macao, and Taiwan were assigned to China. And these studies from England, Northern Ireland, Scotland, and Wales were subsumed into the UK. The detailed information of journals including impact factor (IF), JCR Quartile was obtained from the 2020 Journal Citation Reports (http://clarivate.com/products/web-of-science). H-index means that h articles from one country or author have been cited for at least h times.

### 2.3 Statistical analysis

Statistical analyses were conducted by Microsoft Excel 2019 and R software (v3.6.3.). The growth rate of publications was calculated using the following formula: ((number of publications in the last year ÷ number of publications in the first year)^1/(last year - first year)^ - 1) × 100 (34). Curve fitting was carried out using Microsoft Excel. The model with the highest correlation coefficient (R^2^) value was considered to be the best fitting one. Pearson’s correlation coefficient was utilized to analyze the correlation between two variables. Correlations were considered significant when the correlation *p*-value was < 0.05.

### 2.4 Bibliometric analysis

Data visualization and bibliometric analysis were performed using VOSviewer 1.6.16 (Leiden University, the Netherlands), CiteSpace V 5.8.R3 (Drexel University, the USA), “bibliometrix” package of R software and one online analysis platform (https://bibliometric.com).

VOSviewer, developed by van Eck and Waltman, is a software tool widely used in bibliometric analysis (26). It has a powerful function for constructing and visualizing bibliometric networks such as the co-authorship, co-citation, and co-occurrence network maps of journals, countries, authors, or keywords (35). And these visualization maps were displayed with three different types including the network visualization map, overlay visualization map, and density visualization map. In the current study, VOSviewer was used to perform 1) co-authorship networks of countries, institutions, and authors; 2) co-citation networks of authors and journals; 3) co-occurrence network of keywords; 4) citation analysis of documents. Overall, different visualization maps with different nodes, links and colors represent different meanings. For a more detailed description of these maps, we have provided explanations in the figure legends.

CiteSpace, a free Java-based application founded by Professor Chaomei Chen, is another information visualization software for bibliometric analysis (25). This software is adept at finding key points, especially intellectual pivotal points or turning points, in the development of a certain field and forecasting the research trends by data mining. In this study, it was used to 1) identify keywords/references with strongest citation burst during a certain period; 2) cooperative network of institutions; 3) co-citation networks of references. Moreover, one unique function of this software is to construct the dual-map overlay of journals, which is generated in the context of 10000 journals indexed in the WoSCC. The applied parameters of CiteSpace were set as follows: time slicing (2000/01/01 to 2022/5/28), years per slice (1), g-index (k = 25), link retaining factor (LRF = 3) and selection criteria (top 50 objects).

Additionally, Bibliometrix R-package was used to summarize top 5 authors’ production over time. One online analytical platform was used to perform inter-country collaborative analysis. Moreover, we also searched ClinicalTrials.gov (https://clinicaltrials.gov/) to identify ongoing or completed clinical trials on oncolytic virotherapy for the treatment of central nervous system tumors.

### 2.5 Research ethics

No ethical approval is required since the data used in this study were already in the public domain and did not involve animals/human samples.

## 3 Results

### 3.1 Publication output and temporal trend

**Figure 3:**
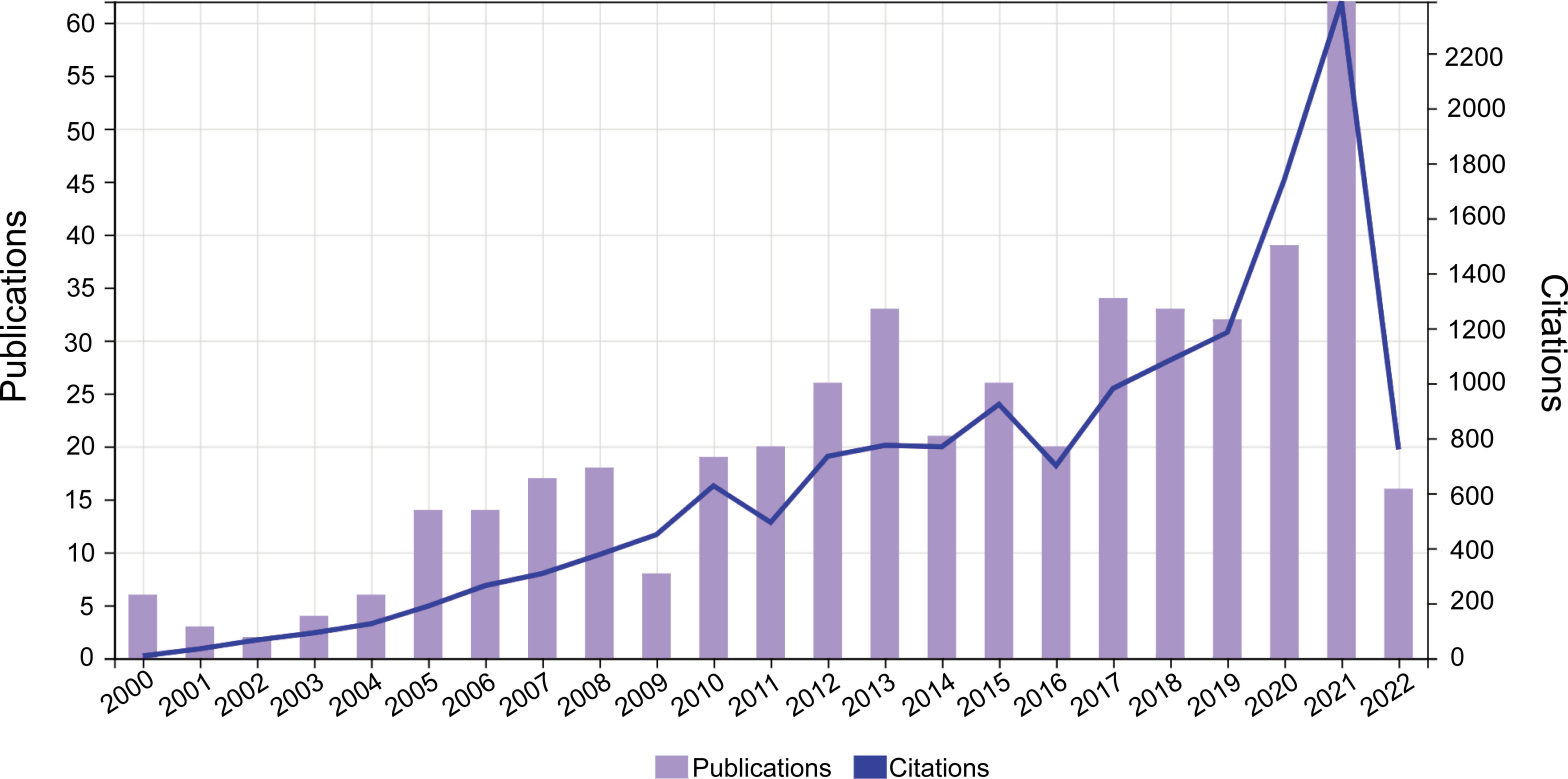
Annual number of publications and citations worldwide from 2000 to 2022.

A total of 473 qualified publications including 341 articles and 132 reviews were finally identified according to the aforementioned retrieval strategy. The annual number of global publications and citations from 2000 to 2022 was presented in **Figure 3**. It can be observed that the annual number of publications related to OVT-CNSTs showed a fluctuating upward trend during the investigated period. The number of documents has increased from 8 in 2000 to 62 in 2021, and the average growth rate was 11.8%. By fitting the data, a statistically significant relationship between the year and output was observed (R^2^ = 0.8281) (**Supplementary Figure 1**). When it comes to citations, all the studies have been cumulatively cited 15071 times with an H-index of 63, and each document was cited 31.86 times on average. Similar to the change tendency of annual output, there was also an ascending trend in the number of citations received by these papers. Pearson correlation analysis demonstrated that the number of publications was positively correlated with citations with a satisfactory Pearson’s correlation coefficient (*r* = 0.949, *p* < 0.001).

### 3.2 Journal distribution and research areas

**Figure 4:**
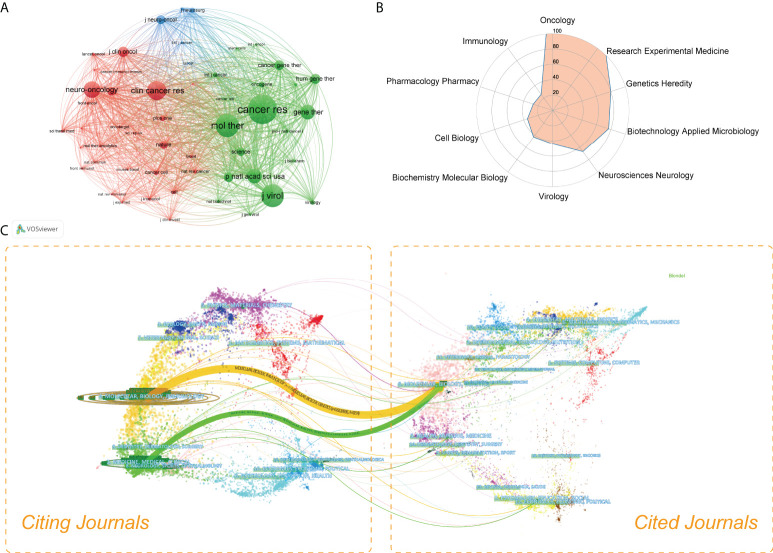
**(A)** Network visualization map of co-cited journals generated by VOSviewer. The nodes represented journals and the links between nodes represented co-citation relationships. The larger the node, the more citations were acquired. **(B)** The radar chart of the top 10 most active research areas in this field. **(C)** The dual-map overlay of OVT-CNSTs research conducted by CiteSpace. In this map, the cited journals appeared on the right side, while the citing journals were on the left side. The wider lines represented the main citing pathways.

All studies in this domain were published in more than 150 different journals. **Table 2** showed the specific information of the top 20 journals ranked by number of publications. Of them, *Molecular Therapy* (22, 4.65%) had published the most studies, followed by *Cancer Gene Therapy* (21, 4.44%) and *Cancers* (19, 4.02%). *Journal of the National Cancer Institute* had the largest impact factor of 13.506. As shown in **Figure 4A**, a network visualization map of journal co-citation analysis was generated by VOSviewer software. A total of 53 journals with a minimum of 100 citations were included. The top 5 most co-cited journals with the largest total link strength (TLS) were *Cancer Research*, *Molecular Therapy*, *Journal of Virology*, *Clinical Cancer Research*, and *Neuro Oncology*. A radar chart of the top 10 most active research areas was illustrated in **Figure 4B**. One can see that *Oncology*, *Research Experimental Medicine*, *Genetics Heredity*, *Biotechnology Applied Microbiology*, and *Neurosciences Neurology* were the top 5 research areas received the most attention. As shown in **Figure 4C**, a dual-map overlay of journals was also created. The wider paths marked in different colors represented the citation relationship of highly active research fields.

**Table 2:** Top 20 most active journals associated with OVT-CNSTs.

Ranking	Journal Title	Publications, n	% of 473	IF 2020	JCR Quartile 2020
1	*Molecular Therapy*	22	4.65	11.454	Q1/Q1/Q1
2	*Cancer Gene Therapy*	21	4.44	5.987	Q1/Q1/Q1/Q2
3	*Cancers*	19	4.02	6.639	Q1
4	*Cancer Research*	18	3.81	12.701	Q1
5	*Journal of Virology*	17	3.59	5.103	Q1
6	*Clinical Cancer Research*	15	3.17	12.531	Q1
7	*Neuro Oncology*	15	3.17	12.3	Q1/Q1
8	*Molecular Therapy Oncolytics*	14	2.96	7.2	Q1/Q1
9	*Gene Therapy*	11	2.33	5.25	Q2/Q1/Q1/Q2
10	*Viruses Basel*	11	2.33	5.048	Q2
11	*Neurosurgical Focus*	8	1.69	4.047	Q2/Q1
12	*PLOS One*	8	1.69	3.24	Q2
13	*Frontiers In Immunology*	7	1.48	7.561	Q1
14	*Frontiers In Oncology*	7	1.48	6.244	Q2
15	*Human Gene Therapy*	7	1.48	5.695	Q1/Q1/Q2
16	*Journal of the National Cancer Institute*	7	1.48	13.506	Q1
17	*Journal of Neuro Oncology*	7	1.48	4.13	Q2/Q3
18	*International Journal of Cancer*	6	1.27	7.396	Q1
19	*Oncogene*	6	1.27	9.867	Q1/Q1/Q1/Q1
20	*Scientific Reports*	6	1.27	4.38	Q1

Ranking: according to the number of total publications.

### 3.3 Global contribution of countries, institutions, authors to the field

#### 3.3.1 Countries/regions

**Figure 5:**
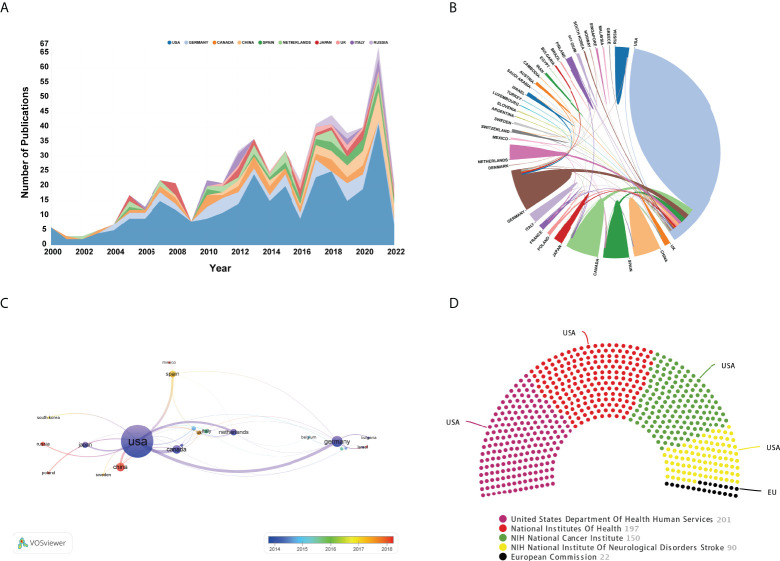
**(A)** Annual number of publications of the top 10 most prolific countries. **(B)** Cooperation of countries/regions. The links between countries/regions represented cooperative relationships and a thicker line indicated a stronger cooperation. **(C)** Overlay visualization map of countries/regions co-authorship analysis conducted by VOSviewer. The nodes represented countries/regions and the links between nodes indicated link strength of a co-authorship relation. The larger the node, the more documents were published by the country. Node color reflected the corresponding average appearing year (AAY) of each country. Based on the color gradient in the lower right corner, red represented the relatively recently published countries, while blue represented the earlier published countries. **(D)** The top 5 funding bodies sponsored the highest number of studies in this domain.

During 2000-2022, scholars from 39 countries/regions have published OVT-CNSTs-related research. The top 15 most prolific countries were listed in **Table 3**. Among them, most were published by scientists from the United States (n=307, 64.9% of all papers) followed by Germany (n=55, 11.63%) and Canada (n=38, 8.03%). The United States has the highest H-index of 57, and the highest citation frequency of 11113 times, which was much higher than that of other countries. From the annual number of outputs in the top 10 most prolific countries, it can be seen that the United States has occupied the dominant position for a long time (**Figure 5A**). **Figure 5B** showed a visualization map of collaboration analysis among countries/regions. We could intuitively observe that Canada was the country with which the United States collaborated most closely, followed by Germany and Spain. As shown in **Figure 5C**, an overlay visualization map of countries/regions co-authorship analysis was generated by VOSviewer. Countries/regions with the minimum number of 3 documents were included. In this map, node color reflected the corresponding average appearing year (AAY) of each country. According to the color gradient in the lower right corner, China, Russia, and Mexico were relatively new entrants compared with these countries marked with blue color such as the United States, Canada, and Germany. As for the top 5 funding agencies for support of OVT-CNSTs research (**Figure 5D**), the United States Department of Health and Human Services (HHS) funded the most studies (201, 42.5%).

**Table 3:** Top 15 most prolific countries in the field of OVT-CNSTs.

Ranking	Countries	Publications, n	% of 473	H-index	TC	AC
1	USA	307	64.90	57	11113	36.2
2	Germany	55	11.63	22	1493	27.15
3	Canada	38	8.03	23	1737	45.71
4	China	37	7.82	13	632	17.08
5	Spain	26	5.50	12	550	21.15
6	Netherlands	25	5.29	14	732	29.28
7	Japan	22	4.65	14	841	38.23
8	UK	14	2.96	10	548	39.14
9	Italy	14	2.96	8	177	12.64
10	France	13	2.75	8	260	20
11	Russia	13	2.75	4	72	5.65
12	Switzerland	9	1.90	7	141	15.67
13	Austria	7	1.48	6	225	32.14
14	Finland	7	1.48	7	317	45.29
15	Sweden	6	1.27	5	151	25.17

Ranking: according to the number of total publications; TC, Total citations; AC, Average citations per item.

#### 3.3.2 Institutions

**Figure 6:**
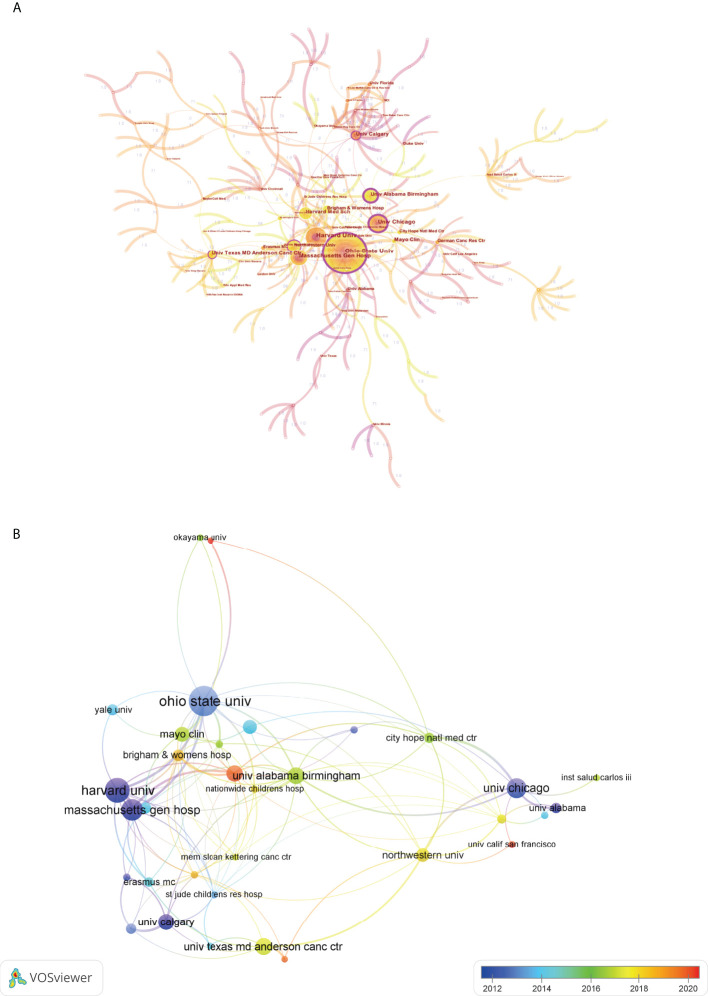
**(A)** Cooperation network map of institutions by using CiteSpace. The nodes represented institutions and the lines mean cooperative relationships between them. The node size was proportional to the publication number. The nodes with outermost purple circle indicated a higher centrality. **(B)** Overlay visualization map of institution co-authorship analysis by using VOSviewer. The meaning of colored lines and nodes was similar to **Figure 5C**.

Institutional collaboration analysis was conducted by CiteSpace. As shown in **Figure 6A**, each node represented an institution, and lines connecting the nodes represented the cooperation relationship. The sizes of the nodes were proportional to the number of documents. It is not difficult to see that the Ohio State University, Harvard University, Massachusetts General Hospital, University of Chicago, and University of Alabama Birmingham were the top 5 institutions with the highest number of publications. Additionally, in this map, these nodes with outermost purple circle indicated a higher centrality, or in other words, the betweenness centrality (BC) value of these nodes were more than 0.1. Generally speaking, BC is a key indicator to reflect the node’s centrality in a network. Thus, these institutions such as the Ohio State University, University of Chicago, University of Alabama Birmingham, University of Calgary, etc., have occupied the central positions in the cooperation network. Moreover, we also provided an overlay visualization map of institution co-authorship analysis by VOSviewer (**Figure 6B**). Only institutions with a minimum of 6 publications were included. The cooperative relationship between these institutions and their AAY could also be clearly seen from this figure.

#### 3.3.3 Authors

**Figure 7:**
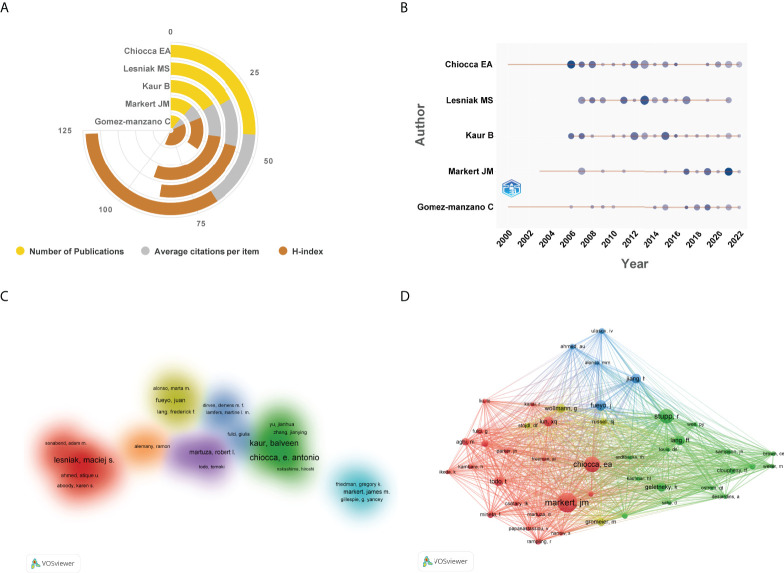
**(A)** The top 5 authors published the highest number of studies in this domain. **(B)** The top 5 authors’ production over time. The circle size represented the number of publications, and the larger the circle, the greater the number of documents. The shade of the circle color signified the total number of citations that the author acquired in this year. The darker the color, the greater the citation times. **(C)** Cluster visualization map of author cooperation analysis. Nodes with close cooperative relationships allocated to the same cluster with the same color. **(D)** Network visualization map of co-cited authors generated by VOSviewer. The meaning of colored lines and nodes was similar to **Figure 3A**.

As for author analysis, the top 5 authors published the highest number of studies were laid out in **Figure 7A**. Of them, Chiocca EA contributed the largest number of documents, followed by Lesniak MS, and Kaur B. The outputs from other authors were no more than 20 documents. Top 5 authors’ production over time was shown in **Figure 7B**. In addition, a cluster visualization map of author cooperation analysis was depicted in **Figure 7C**. Nodes with close cooperative relationships were allocated to the same cluster with the same color. Of them, only 7 author clusters were formed and there was relatively less cooperation between researchers from different institutions. In terms of author co-citation analysis, 45 researchers with at least 50 citations were included in the network map. As displayed in **Figure 7D**, Markert JM has the highest number of co-citations, followed by Chiocca EA, and Stupp R.

### 3.4 Highly cited studies and co-cited references

**Figure 8:**
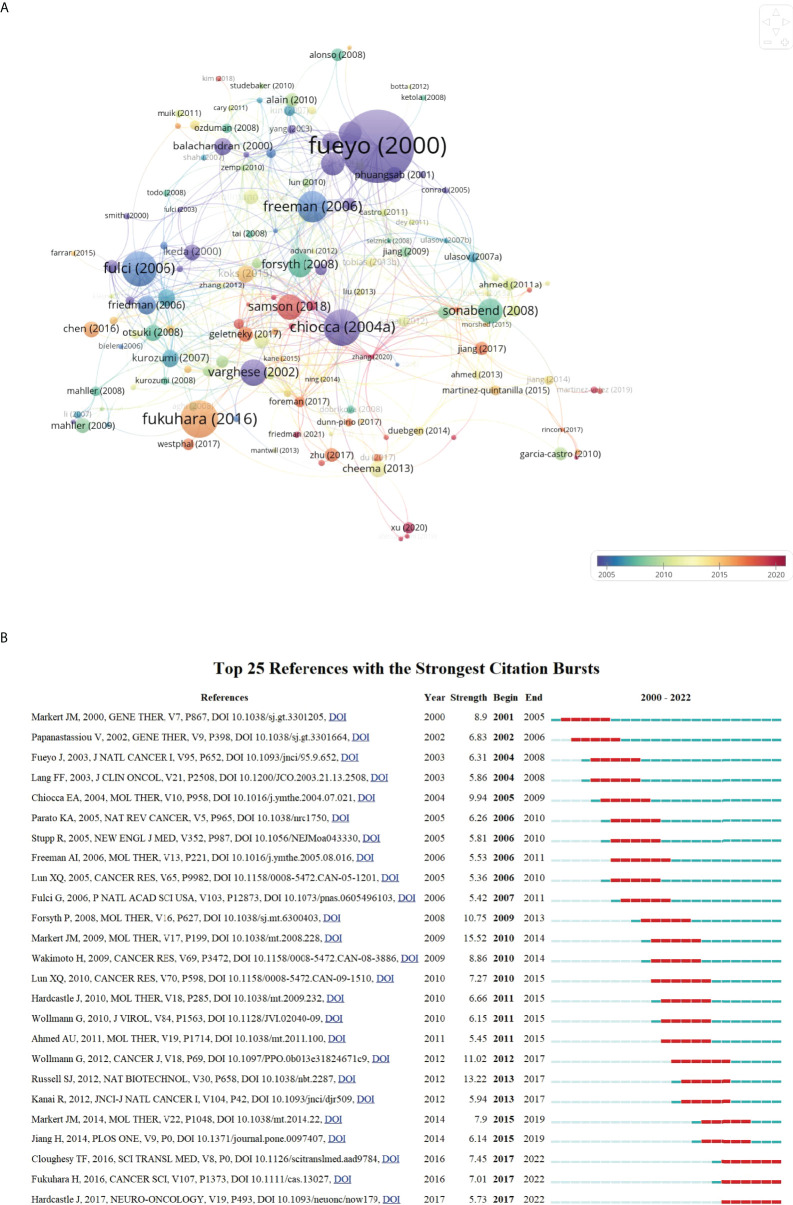
**(A)** Network visualization map of document citation analysis generated by VOSviewer. The node size was proportional to the citation times. **(B)** Top 25 references with the strongest citation bursts. The blue line represented the time axis, and the red bars indicated the burst period.

The network visualization map of document citation analysis was generated by VOSviewer. As can be seen from **Figure 8A**, the node size was proportional to the citation times. The most highly-cited study was written by Fueyo et al. (36) published in *Oncogene*. Along with this, we also summarized the basic information of the top 20 most cited studies in this field in **Table 4**. All of them were published between 2000 and 2018, and four-fifths of which were published prior to 2010. Besides, references analysis is one of the most significant indicators in bibliometrics. Co-cited references refer to references that have been cited together by other documents. As shown in **Supplementary Figure 2**, all the references were clustered into 20 main sub-clusters. The silhouette value of these clusters was all more than 0.89 (0.896-1) with a mean value of 0.9418, indicating the good homogeneity of these clusters.

**Table 4:** Top 20 highly cited studies in OVT-CNSTs field.

Ranking	Title	TC	AC	Journal	First Author	Publishedyear
1	A mutant oncolytic adenovirus targeting the Rb pathway produces anti-glioma effect *in vivo*	580	25.22	*Oncogene*	Fueyo, J	2000
2	Oncolytic virus therapy: A new era of cancer treatment at dawn	286	40.86	*Cancer Science*	Fukuhara, Hiroshi	2016
3	A phase I open-label, dose-escalation, multi-institutional trial of injection with an E1B-attenuated adenovirus, ONYX-015, into the peritumoral region of recurrent malignant gliomas, in the adjuvant setting	281	14.79	*Molecular Therapy*	Chiocca, EA	2004
4	Cyclophosphamide enhances glioma virotherapy by inhibiting innate immune responses	265	15.59	*Proceedings Of the National Academy of Sciences of The United States of America*	Fulci, Giulia	2006
5	Phase I/II trial of intravenous NDV-HUJ oncolytic virus in recurrent glioblastoma multiforme	237	13.94	*Molecular Therapy*	Freeman, AI	2006
6	Oncolytic herpes simplex virus vectors for cancer virotherapy	198	9.43	*Cancer Gene Therapy*	Varghese, S	2002
7	Intravenous delivery of oncolytic reovirus to brain tumor patients immunologically primes for subsequent checkpoint blockade	189	37.8	*Science Translational Medicine*	Samson, Adel	2018
8	Mesenchymal stem cells effectively deliver an oncolytic adenovirus to intracranial glioma	188	12.53	*Stem Cells*	Sonabend, Adam M	2008
9	A phase I trial of intratumoral administration of reovirus in patients with histologically confirmed recurrent malignant gliomas	182	12.13	*Molecular Therapy*	Forsyth, Peter	2008
10	Armed Oncolytic Virus Enhances Immune Functions of Chimeric Antigen Receptor-Modified T Cells in Solid Tumors	181	20.11	*Cancer Research*	Nishio, Nobuhiro	2014
11	Reovirus as an oncolytic agent against experimental human malignant gliomas	173	7.86	*Journal Of the National Cancer Institute*	Wilcox, ME	2001
12	Targeted molecular therapy of GBM	169	8.45	*Brain Pathology*	Mischel, PS	2003
13	Glioma virotherapy: Effects of innate immune suppression and increased viral replication capacity	136	8	*Cancer Research*	Friedman, A	2006
14	Vesicular stomatitis virus (VSV) therapy of tumors	130	5.65	*Iubmb Life*	Balachandran, S	2000
15	Depletion of peripheral macrophages and brain microglia increases brain tumor titers of oncolytic viruses	129	8.06	*Cancer Research*	Fulci, Giulia	2007
16	Effect of tumor microenvironment modulation on the efficacy of oncolytic virus therapy	127	7.94	*JNCI-Journal of the National Cancer Institute*	Kurozumi, Kazuhiko	2007
17	Myxoma virus is a novel oncolytic virus with significant antitumor activity against experimental human gliomas	127	7.06	*Cancer Research*	Lun, XQ	2005
18	Newcastle disease virotherapy induces long-term survival and tumor-specific immune memory in orthotopic glioma through the induction of immunogenic cell death	124	15.5	*International Journal of Cancer*	Koks, Carolien A	2015
19	Multifaceted oncolytic virus therapy for glioblastoma in an immunocompetent cancer stem cell model	122	12.2	*Proceedings of the National Academy of Sciences of the United States of America*	Cheema, Tooba A	2013
20	Histone deacetylase inhibitors augment antitumor efficacy of herpes-based oncolytic viruses	121	8.07	*Molecular Therapy*	Otsuki, Akihiro	2008

Ranking: according to the number of total citations.

### 3.5 References burst analysis

To better understand the developmental process of OVT-CNSTs research in the past years, references with strong citation bursts were identified by CiteSpace. **Figure 8B** displayed the top 25 references with the strongest citation bursts from 2000 to 2022. Among them, reference with citation burst first emerged in 2001 owing to one study published in 2000. These studies by “Markert et al in 2009”, “Russell et al. in 2012” and “Wollmann et al in 2012” were the top 3 references with the strongest burst strength (15.52, 13.22, and 11.02, respectively) (37–39). Of note, there were three most recent references with citation bursts beginning from 2017, and the bursts are still ongoing.

### 3.6 Keywords co-occurrence analysis

**Figure 9:**
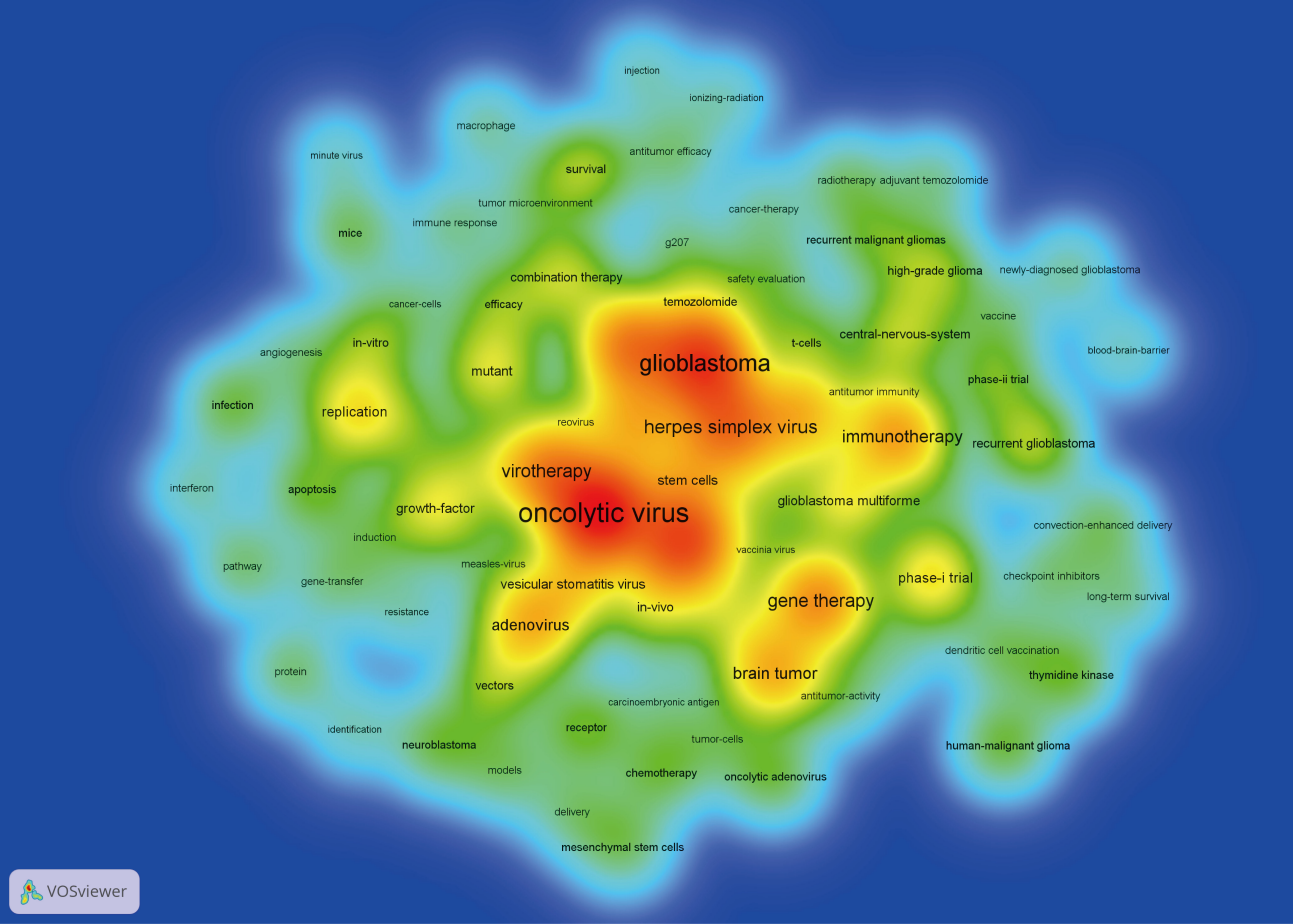
Keyword co-occurrence analysis by VOSviewer. In this density visualization map, the deeper the color, the higher the co-occurrence frequency of keywords.

**Figure 10:**
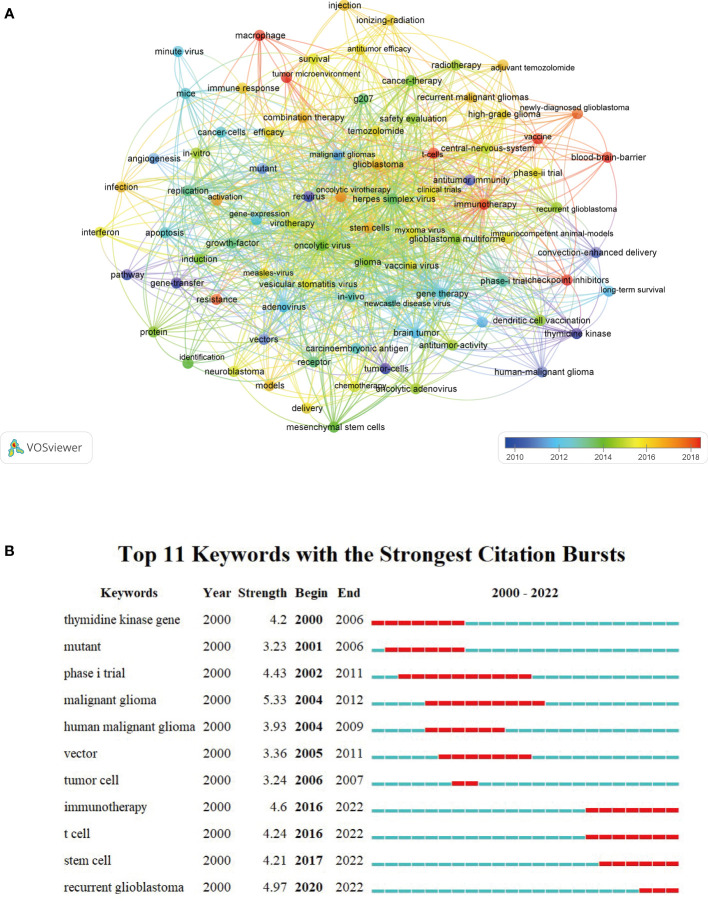
**(A)** Overlay visualization map of keyword co-occurrence analysis by VOSviewer. Each node represented a keyword. The node size was proportional to the occurrence frequencies. Node color reflected the corresponding AAY of each keyword (based on the color gradient in the lower right corner). **(B)** Top 11 keywords with the strongest citation bursts by CiteSpace. The blue line represented the time axis, and the red bars indicated the burst period.

A density visualization map of keywords was shown in **Figure 9**. After merging the keywords with the same meaning manually, a total of 85 keywords with a minimum of 10 occurrences were extracted from the 473 publications. Among them, the top 20 most frequent keywords associated with OVT-CNSTs was summarized in **Table 5**. Keywords with the highest occurrence frequencies were as follows: “oncolytic virus” (179), “glioblastoma” (140), “glioma” (130), “malignant gliomas” (95), “herpes simplex virus” (92), “gene therapy” (86), “virotherapy” (83), “immunotherapy” (73), “brain tumor” (68), and “adenovirus” (63). Furthermore, we also provided an overlay visualization map of these keywords in **Figure 10A**. According to the color gradient in the lower right corner, keywords with a relatively latest AAY such as “immunotherapy”, “T-cells”, “tumor microenvironment”, “vaccine”, “blood-brain-barrier”, “checkpoint inhibitors” and “macrophage”, were the research focuses in recent years.

**Table 5:** Top 20 most frequent keywords associated with OVT-CNSTs.

Ranking	Keywords	Occurrences	AAY	Ranking	Keywords	Occurrences	AAY
1	oncolytic virus	179	2014.19	11	clinical trials	62	2015.48
2	glioblastoma	140	2016.49	12	oncolytic virotherapy	58	2017.02
3	glioma	130	2014.47	13	phase-I trial	50	2013.00
4	malignant gliomas	95	2011.53	14	replication	46	2013.28
5	herpes simplex virus	92	2013.64	15	stem cells	43	2016.57
6	gene therapy	86	2012.65	16	vesicular stomatitis virus	42	2015.48
7	virotherapy	83	2015.02	17	glioblastoma multiforme	41	2014.46
8	immunotherapy	73	2017.93	18	growth-factor	39	2013.41
9	brain tumor	68	2011.84	19	mutant	39	2011.23
10	adenovirus	63	2011.94	20	combination therapy	33	2016.50

AAY, average appearing year.

### 3.7 Keywords burst analysis

In the present study, we applied the burst detection algorithm to extract keywords from OVT-CNSTs-related publications. A total of 11 keywords with the strongest citation bursts were identified (**Figure 10B**). Of them, “malignant glioma”, “recurrent glioblastoma” and “immunotherapy” were the top 3 keywords with the strongest burst strength (5.33, 4.97, and 4.6, respectively). Remarkably, the citation bursts time of these keywords including “immunotherapy” (2016-2022), “T cell” (2016-2022), “stem cell” (2017-2022), and “recurrent glioblastoma” (2020-2022) has continued to 2022 and the bursts are still ongoing.

## 4 Discussion

### 4.1 The global research dynamics in the field of OVT-CNSTs

In the present study, we chose publications indexed in WOSCC as the data source, and multiple bibliometric tools were used to construct and visualize the bibliometric networks of literature related to OVT-CNSTs. In order to elucidate the global research trends in different developmental stages from 2000 to 2022, preliminary analyses were conducted to observe the annual number of publications and citations regarding OVT-CNSTs. As can be seen from the result in **Figure 3**, there is a general upward trend in the number of OVT-CNSTs-related publications in the past years. In particular, studies published in the last five years (2018-2022) accounted for 38.5% of all the publications for the whole period, indicating that this topic has received substantial attention in recent years. One key inflection point occurred in the year 2021, which might be associated with several significant events in the field of OVT-CNSTs. Undoubtedly, the most remarkable event was the first approval of genetically engineered oncolytic virus, G47Δ, for the treatment of malignant gliomas, ushering in a new era of oncolytic virotherapy for central nervous system tumors (17). Therefore, we boldly predict that this field will continue to progress at a rapid pace in the near future.

### 4.2 The most popular and influential journals in this domain

Journal analysis is able to assist researchers accurately grasp the core journals in their research fields and quickly find the suitable journals for literature queries, data collection, and manuscript submission (29, 40, 41). Therefore, we have summarized the detailed information of the top 20 active journals in this field. As displayed in **Table 2**, many of these journals belong to the categories of oncology, virology, biotechnology and applied microbiology. *Molecular Therapy*, *Cancer Gene Therapy*, *Cancers*, *Cancer Research*, and *Journal of Virology* were the most popular journals published relatively larger number of studies in this area. This result also suggested that these journals were particularly interested in works regarding OVT-CNSTs. In addition, according to the JCR 2020 standards, almost all of these top 20 journals were classified as Q1 or Q2, indicating that these journals possess high academic impact. It is generally presumed that studies published in high-impact journals tend to have an extensive popularity among scholars and thus more likely to be cited by other researchers, which in turn maintains the high impact factor of these journals. From the results of journal co-citation analysis, we can see that these journals including *Cancer Research*, *Molecular Therapy*, *Journal of Virology*, *Clinical Cancer Research*, and *Neuro Oncology* were also the most co-cited journals in this field. Therefore, it could be envisaged that future major developments within this field are more likely to be published in the aforementioned journals.

Results from **Figure 4B** revealed that *Oncology*, *Research Experimental Medicine*, *Genetics Heredity*, *Biotechnology Applied Microbiology*, and *Neurosciences Neurology* were the top 5 popular research categories received most attention in the field of OVT-CNSTs. It was not difficult to see that research on oncolytic virotherapy for treatment of central nervous system tumors has grown as a multidisciplinary area that integrated knowledges from several clinical and basic disciplines. Additionally, dual-map is often used to depict knowledge flow and citation pattern in a certain field (5). As is evident from this map, there were two core citation paths with orange or green color. These citation paths implied that majority of studies published in journals of Molecular/Biology/Immunology and Medicine/Medical/Clinical were likely to be biased to cite literature published in journals belonging to the field of Molecular/Biology/Genetics. This result also indicated that the current oncolytic virotherapy research for central nervous system tumors has focused largely on basic research and translational medicine.

### 4.3 The main contributors from the level of countries, institutions, authors

In general, the number of publications is an important indicator to mirror the contribution of a country, an institution or an author in a certain field. Whether it is from the perspective of outputs or quality, there is no doubt that the United States has played the most important role in the field of OVT-CNSTs research in the past decades. Of note, multiple previous bibliometric studies have also confirmed the leading edge of the United States in the area of brain tumors (28, 42). As for the reasons, it is commonly deemed that high-quality research is inseparable from the substantial investment from various funding bodies and governments. Central nervous system tumor represents an important cause of cancer-related morbidity and mortality in the United States. To address this challenge, the US government has invested tremendous resources in this field, and an increasing number of researchers has devoted themselves to CNSTs-related studies. In 1994, spurred by the complexity of CNSTs and urgent need for effective treatment strategies, the United States National Cancer Institute (NCI) dedicated one specialized committee consisting of top neuro-oncologists to strive for novel therapeutic approaches of brain tumors (43). As can be seen from the distribution of the top 5 most frequent funding sources in this field, more than half of them were from the United States. This may be an important reason why the United States has the highest research strength and academic impact in this area. Furthermore, the existence of top academic institutions and scientists may be another key factor. It is not difficult to see that the top 5 institutions, that had the highest number of publications including Ohio State University, Harvard University, Massachusetts General Hospital, University of Chicago, and University of Alabama Birmingham, were all located in the United States. In terms of institutional collaboration analysis, majority of the institutions with the highest BC values were resident in the United States. Once again, it demonstrated the global leadership of the United States on OVT-CNSTs research.

As for author contribution analysis, **Figures 7A, B** showed the top 5 productive authors in this domain. As expected, all these researchers were from the United States. Specifically, Chiocca EA from Brigham and Women’s Hospital, Lesniak MS from Feinberg School of Medicine, and Kaur B from University of Texas Health Science Center Houston contributed the highest number of documents (more than 20 papers). Professor Chiocca’s team is mainly focused on the preclinical evaluation of oncolytic viruses, especially recombinant herpes virus for glioma therapy, and multiple methods to enhance antitumor innate immune response of viral oncolytic immunotherapy to brain tumors (44–46). One phase I open-label, dose-escalation, multi-institutional trial conducted by his team and collaborators has garnered a significant amount of attention. Their findings suggested that injection of ONYX-015, an E1B-attenuated adenovirus, into brain surrounding a resected malignant glioma (24 patients) was well tolerated and relative safety at doses up to 10^10^ plaque-forming units (15). While Lesniak MS mainly focuses on stem cell-based carriers such as neural stem cells or mesenchymal stem cells to deliver oncolytic adenovirus for malignant gliomas (47–49). As for author co-citation analysis, Markert JM has the highest number of co-citations, followed by Chiocca EA, and Stupp R. These authors have been co-cited more than 190 times, indicating that their studies on oncolytic virotherapy for central nervous system tumors have received widespread attention (50–52).

Moreover, it is worth noting that from the results of collaboration analysis, there is little cooperation among institutions and authors from different countries, and most of the studies were conducted at a single institution or team. Since CNST is a common threat to the whole humanity, future researchers should strengthen more comprehensive and extensive cooperation between different countries to boost the development of OVT-CNSTs research (53).

### 4.4 The main research directions and focuses currently

In general, citation analysis is considered as an important metric to evaluate the relative academic impact of one document in a certain field. Highly cited articles are not only identified as excellent scientific works, but also revealed hot directions, which has been attracting substantial research attention. As can be seen from the top 20 highly cited in this field, three of them were published as review articles and the remaining 17 were published as research articles. Of these reviews, they have summarized different classes of oncolytic virus including genetically engineered and naturally occurring oncolytic viruses, their mechanisms of oncolysis and clinical trial outcomes (11, 18, 54). These reviews contribute to the understanding of the existing knowledge about OVT-CNSTs and could be considered as the entry-level reads for young scholars. Besides that, the remaining articles mainly focus on the preclinical and clinical results of different oncolytic virus for treating central nervous system tumors. Of them, one study conducted by Fueyo et al., had the highest number of citations (36). In this study, they constructed a tumor-selective adenovirus (Delta24) with a deletion of 24 base pairs in the E1A region, that prevented the binding of E1A to Rb protein. Thus, this virus was not able to release E2F and replicate in normal cells, but could be useful against gliomas and other cancers with disrupted Rb pathway. Additionally, there were also multiple studies reported the results of both *in vitro* and *in vivo* antitumor effects of various oncolytic viruses such as reovirus (55), vesicular stomatitis virus (56), newcastle disease virus (57), G47Δ-mIL12 (58) as well as myxoma virus (59), against central nervous system tumors and explored possible oncolytic mechanisms such as inducing immunogenic cell death, targeting GSCs and tumor microenvironment, and so on. Along with this, several studies proposed multiple strategies to boost oncolytic efficacy for CNSTs, including valproic acid or cyclophosphamide pretreatment (46, 60), a combination with angiostatic cRGD peptide (61), addition of immune checkpoint inhibitors (ICIs) (62) or chimeric antigen receptors (CARs) (63). However, not all patients could benefit significantly from combination therapies. One phase Ib randomized study has evaluated the clinical effects and safety of combination therapy with intra-tumoral DNX-2401 (a replication-competent, tumor-selective and oncolytic adenovirus) plus interferon-γ on glioblastoma patients. The result showed that patients receiving this combinatory regimen were poorly tolerated and showed no additional clinical benefit over DNX-2401 alone (64).

Meanwhile, three studies have introduced the phase I/II trial results of ONYX-015 (E1B-Attenuated adenovirus), NDV-HUJ (the oncolytic HUJ strain of Newcastle disease virus), and reovirus for the treatment of patients with recurrent malignant gliomas (15, 65, 66). To further explore the research interest of scholars in current clinical studies on OVT-CNSTs, we summarized the relevant clinical studies in this field. As summarized in **Supplementary Table 1** and **Supplementary Table 2**, there were 32 clinical trials including 23 in adults and 9 in children. Among them, we found the highest number of OVT-CNSTs clinical trials has completed or is going on in the United States, most of which were in phase I. The method of these studies was mainly interventional. Among the various oncolytic viruses, the number of clinical trials involving Herpes Simplex Virus (OH2, G207, M032, C134, etc.) is the highest. Collectively, although there are still many unsolved issues on oncolytic virotherapy, such as mode of administration, infectivity efficiency, infection of off-target (nontumor) tissues and safe dose, it has already advanced to clinical trials for treatment of central nervous system tumors and exhibited high antitumor potential in clinical application. Future studies should more comprehensively evaluate the myriad oncolytic virus vectors under development to determine which would be more appropriate for clinical translation.

In addition to highly cited studies, references analysis of all the publications could also reflect the main research directions and emerging trend of a specific field to some extent. By using CiteSpace, these references with sharp increases of attention within a certain period have been identified. The top 25 references with the strongest citation bursts from 2000 to 2022 were displayed in **Figure 8B**. In this figure, the blue line represented the time axis, and the red bars indicated the burst period. With citation bursts first emerged in 2001 owing to one study published in 2000 by Markert and colleagues (67). In this study, they sought to determine the safety of G207 (HSV-1 strain F) inoculation into cerebral malignant glial tumors in humans. The radiographic and neuropathologic evidences suggested that there were no toxicity or serious adverse events unequivocally ascribed to G207 and long-term presence of viral DNA in some cases. Notably, although the burst periods in majority of references were over, the bursts of three recent references are ongoing until now, indicating that these studies are still receiving a lot of attention in recent years (18, 68, 69). Of them, apart from the review article mentioned before, one phase I trial reported the safety and efficacy of recurrent high-grade glioma treated with Toca 511 (vocimagene amiretrorepvec) and Toca FC (extended-release 5-fluorocytosine) (68). Another study demonstrated that oncolytic measles virotherapy in combination with anti-PD-1 antibody blockade could significantly enhance antitumor activity in glioblastoma treatment (69). Taken together with the present results, it could be concluded that the current studies in this field mainly focused on the clinical trials and immunovirotherapy of different oncolytic virus for CNSTs treatment.

### 4.5 The most concerned research frontiers and hotspots the in the future

Generally speaking, keywords are the refinement of the core ideas of one article and usually considered as an important index to reflect research frontiers and hotspots in a particular field (4). In this study, we employed keyword co-occurrence analysis to identify the most widely used keywords to reveal key topics within the OVT-CNSTs knowledge base. After merging keywords with the same meaning, a total of 85 keywords with a minimum of 10 occurrences were extracted. Among them, glioma, especially glioblastoma, is the most concerned central nervous system tumor, while HSV, adenovirus, vesicular stomatitis virus are the most studied oncolytic viruses in this field. Moreover, we found that the following research topics including gene therapy, immunotherapy, combination therapy, and clinical trials have also received extensive attention from neuroscientists. As shown in **Figure 10A**, we provided an overlay visualization map of co-occurrence keywords. In this map, different colors were applied for each keyword based on their average appearing year in articles. Keywords appeared relatively earlier were marked with blue or purple color, while those marked with red representing more recent appearance. Overall, these keywords with a relatively latest AAY such as “immunotherapy”, “T-cells”, “tumor microenvironment”, “vaccine”, “blood-brain-barrier”, “checkpoint inhibitors” and “macrophage” were the research focuses of this field in recent years (70). Apart from keyword co-occurrence analysis, keywords with strong burst strength were regarded as another indicator that could help predict new frontier topics. Remarkably, there were four recent keywords including “immunotherapy”, “T cell”, “stem cell” and “recurrent glioblastoma” with ongoing burst until 2022, implying that these topics also have great potential to continue to be hotspots for future research. All in all, by using both VOSviewer and Citespace software, nine promising research directions were identified.

Take immunotherapy as an example, CNS neoplasms, especially glioblastoma, is one of the most immunosuppressive tumors that have high resistance against current available therapies. So far, many clinical trials of immunotherapy including ICIs targeting glioblastoma have not been successful (71, 72). Recently, combinations of oncolytic virotherapy with other immunotherapies such as ICIs, antigen-specific T-cell receptors (TCRs), and CARs have led to tremendous progress in antitumor treatment. In the field of CNSTs, Saha et al., reported the synergistic interaction between G47Δ⁃mIL⁃12, an oncolytic HSV encoding IL-12, and ICIs (anti-PD-1 and anti-CTLA-4) in curing glioblastoma and inducing immunological memory (73). Their findings showed that the combination of G47Δ⁃mIL⁃12 and ICIs could eradicate glioma in two mouse models, which was associated with T cell activation, an influx of tumor-associated macrophages (TAMs) and M1-like polarization, along with increased T effector to T regulatory ratio. Jiang and colleagues constructed an oncolytic adenovirus Delta-24-RGDOX, that expressed the immune costimulator OX40 ligand (OX40L) (74). Then they combined Delta-24-RGDOX with intratumoral injection of a PD-L1 antibody in a glioma mouse model. The results showed that this new tumor-targeting combination strategy induced a synergistic therapeutic effect and exhibited exceptional anti-tumor efficacy. Interestingly, similar results were also obtained in other combination therapeutic studies (62, 63, 69). In addition, the use of viral vectors including replication-deficient viruses, oncolytic viruses, and replicating nonlytic viruses to deliver immunotherapies directly to tumor cells has also been proven to be an effective method to improve targeted delivery to the CNSTs and reduce systemic toxicities (75). To summarize, the above findings highlight that these oncolytic viruses are potential antitumor immunotherapeutic agents and the combination of OVT with other immunotherapy approaches is an attractive strategy for central nervous system tumors therapy in the future (76).

As mentioned earlier, the blood-brain-barrier is a major obstacle that hinders the intravenously administered anticancer agents or genes entering the brain tumor tissue (4, 5). Multiple studies have shown that several oncolytic viruses, especially RNA viruses with relatively small structures, were able to across the BBB and thereby enabled intravenous delivery, which could be the unique advantage of oncolytic virotherapy for CNSTs (77, 78). Despite this, an inefficient extravasation of these viruses from vascular to extravascular compartments has drastically curtailed the virus particles reaching tumor tissue due to this physical barrier. Therefore, in OVT-CNSTs clinical trials, most of oncolytic viruses were being administered locally instead of systemic delivery to circumvent the obstacle of BBB and maximize the virus load in tumors. In recent years, various methods have been proposed to optimize delivery of oncolytic viruses beyond the BBB. For example, several scholars have introduced a method of intranasal delivery to deliver oncolytic virus to glioma bypassing the BBB (79). Moreover, as outlined previously, stem cell-based carriers including neural stem cells or mesenchymal stem cells could also be used to deliver oncolytic adenovirus to malignant gliomas (47–49). During this process, there were also studies using endovascular selective intra-arterial administration technique, an approach disrupting the BBB through hyperosmotic solution, to facilitate localized delivery of stem cells (77, 80). However, most of these approaches are still at an exploratory stage, and future studies can be performed in the preclinical and clinical setting to identify the ideal delivery strategies.

### 4.6 Strengths and limitations

To our knowledge, this is the first work to conduct a comprehensive analysis of global research developments and hotspots in the field of OVT-CNSTs. Compared with previous bibliometric reports, a strength of this study is the use of multiple bibliometric tools that could help integrate information and make the analysis more comprehensive and credible. Nevertheless, there are still deficiencies in the present study. Firstly, as several studies mentioned, we only chose WoSCC to search the related publications, which may lead to incomplete retrieval of literature. However, it is commonly accepted that different databases have different properties such as document type marking, citations counting, and export formats, thus incorporation analysis of multiple databases may not the optimal choice for bibliometric studies (4). Most of previous studies were only based one database, among which WoSCC was the most commonly used one (32, 81). Secondly, only studies published in English were enrolled, which means several potential studies with other languages could be missed. Thirdly, although the retrieve strategy was enriched as much as we can, several related documents and clinical trials regarding OVT-CNSTs might be omitted. Regardless of these limitations, we are confident that our study could reflect the research trends and hotspots in the field of OVT-CNSTs.

## 5 Conclusion

In conclusion, the field of OVT for the treatment of CNSTs is continuously evolving and expanding over the past two decades. The potential of this therapeutic strategy is being recognized in increasing numbers of studies. We comprehensively analyze scientific publications related to OVT-CNSTs from various perspectives such as global research trends, active journals, main contributors. More importantly, the main research directions and focuses currently, and the most concerned research frontiers and hotspots in the future were also summarized in detail. All in all, our bibliometric study provides an essential reference for later researchers to better understand the basic knowledge landscapes, find potential collaboration opportunities with other research teams, identify the hot research topics currently, and grasp research frontiers in the future.

## Data availability statement

The original contributions presented in the study are included in the article/**Supplementary Material**. Further inquiries can be directed to the corresponding authors.

## Author contributions

KC, QG, PZ, YZ, WY, YW, YL, ZS and HW designed the study. KC, QG, PZ and YZ collected the data. KC, WY, YW and YL analyzed the data and drafted the manuscript. KC, HZ, YL, ZS and HW revised and approved the final version of the manuscript. All authors read and approved the submitted version.

## Acknowledgments

The authors thank Dr. Yuqiao Li of Tianjin Medical University, professor Xiuhua Yao from Tianjin huanhu hospital, and “home-for-researchers (www.home-for-researchers.com)” for their help in in polishing our English writing.

## Conflict of interest

The authors declare that the research was conducted in the absence of any commercial or financial relationships that could be construed as a potential conflict of interest.

## Publisher’s note

All claims expressed in this article are solely those of the authors and do not necessarily represent those of their affiliated organizations, or those of the publisher, the editors and the reviewers. Any product that may be evaluated in this article, or claim that may be made by its manufacturer, is not guaranteed or endorsed by the publisher.

## Abbreviations

CNST, central nervous system tumor; CNSTs, central nervous system tumors; OVT, oncolytic virotherapy; OVT-CNSTs, oncolytic virotherapy for central nervous system tumors; GSCs, glioma stem cells; BBB, blood-brain barrier; T-VEC, talimogine laherparepvec; HSV-1, herpes simplex virus-1; WOSCC, Web of Science Core Collection; IF, impact factors; TLS, total link strength; BC, betweenness centrality; AAY, average appearing year; TCRs, T-cell receptors; ICIs, immune checkpoint inhibitors; CARs, chimeric antigen receptors; TAMs, tumor-associated macrophages.
