# Supplementary material for: Emerging trends and research foci of oncolytic virotherapy for central nervous system tumors: A bibliometric study
Source: Front Immunol. 2022 Sep 6;13:975695. doi: 10.3389/fimmu.2022.975695 (PMC9486718; doi:10.3389/fimmu.2022.975695)
Supplement: Supplementary file 1 [file DataSheet_1.docx]

Supplementary Material

## Supplementary Figures


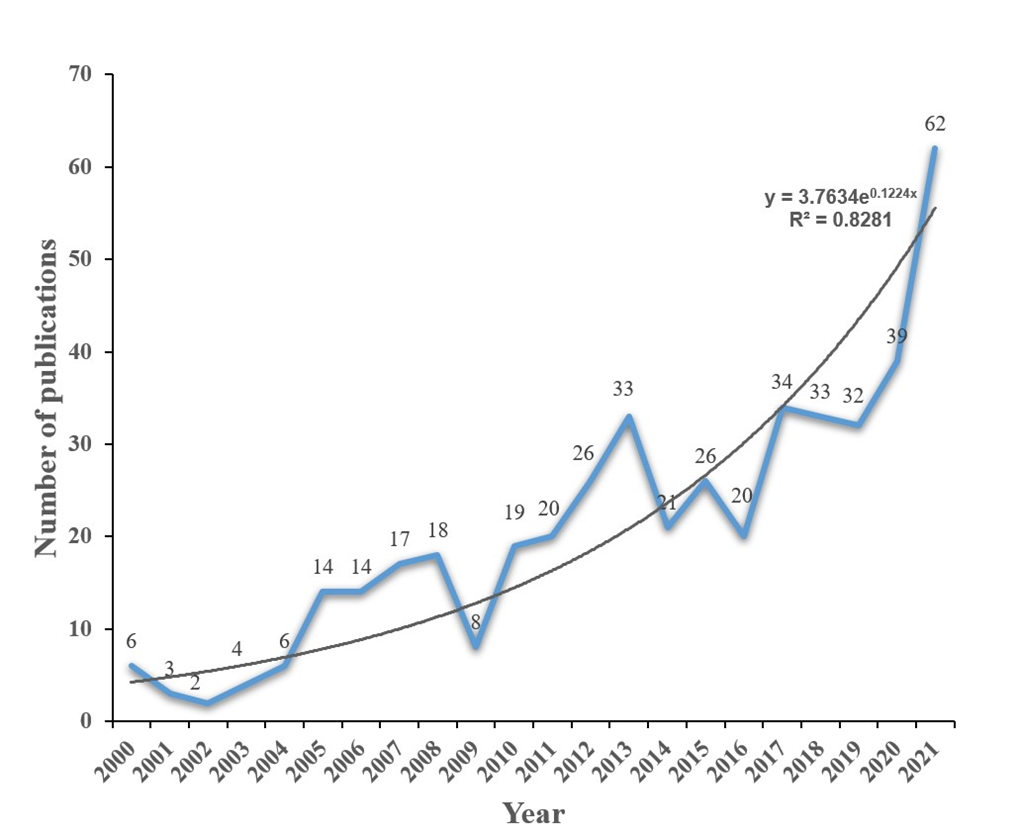


**Supplemental Figure 1. Curve fitting of annual growth trend of publications from 2000 to 2021.**

**
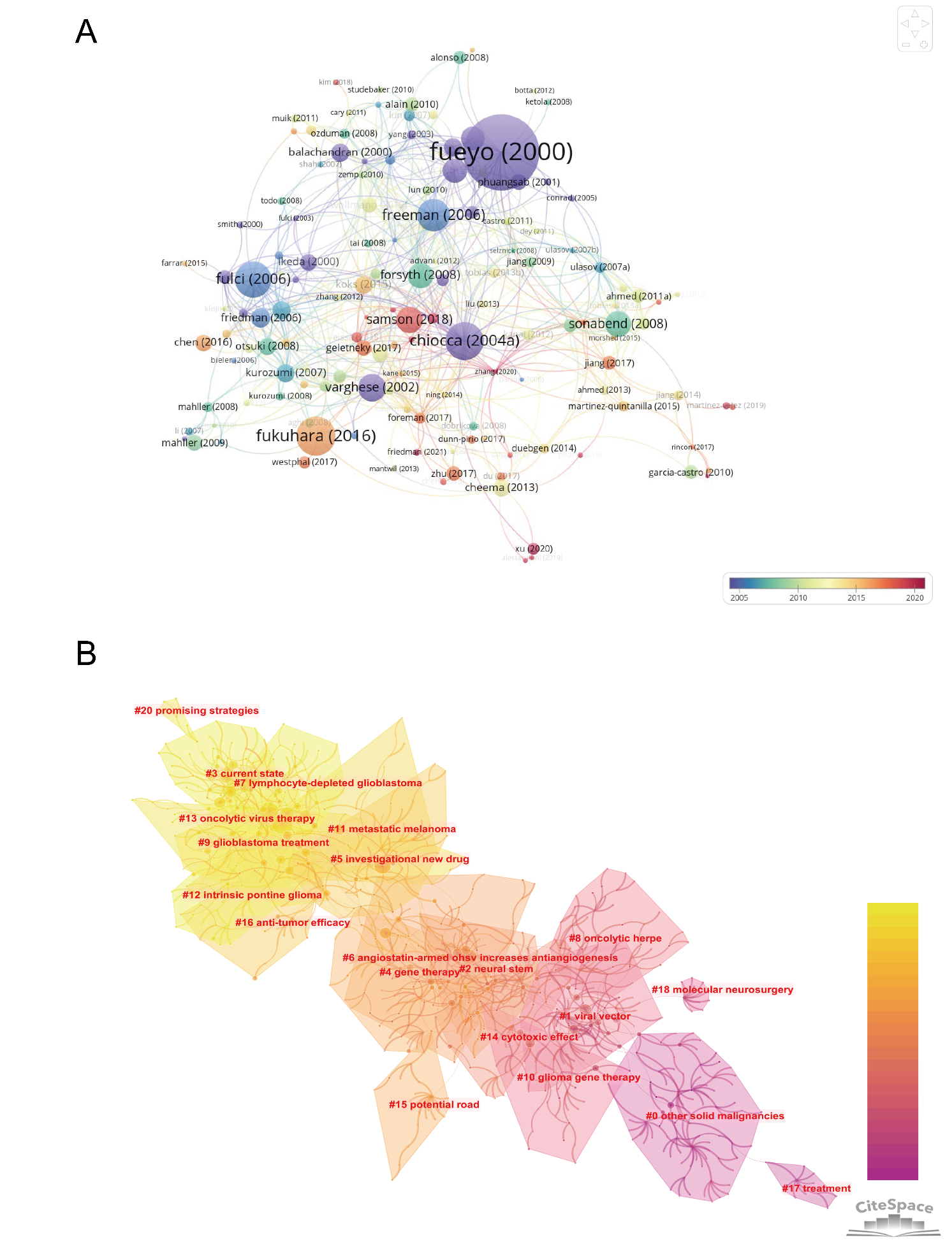
**

**Supplemental Figure 2. Network visualization map of reference co-citation analysis generated by the CiteSpace. All the references are clustered into 20 clusters. From 2000 to 2022, the color changed from deep to light yellow.**

## Supplementary Table

**Supplementary Table 1: Adult clinical studies related to OVT-CNSTs**

| Study | NCT | Office title | Phase | Status | Number Enrolled | Country | Time | Interventions | Summary |
| --- | --- | --- | --- | --- | --- | --- | --- | --- | --- |
| 1 | NCT05235074 | OH2 Oncolytic Viral Therapy in Central Nervous System Tumors | Phase 1/Phase 2 | Recruiting | 28 | China | 2021/11/16-2025/6/15 | Biological: OH2 injection | This study mainly explores the safety, tolerability and preliminary effectiveness of two doses of OH2 injection in the treatment of patients with recurrent central nervous system tumors |
| 2 | NCT03072134 | Neural Stem Cell Based Virotherapy of Newly Diagnosed Malignant Glioma | Phase 1 | Completed | 13 | USA | 2017/4/24-2021/7/1 | Biological: Neural stem cells loaded with an oncolytic adenovirus | The primary objectives are to evaluate the safety of the combined therapy and determine the maximum tolerated dose (MTD) for a future Phase II study. |
| 3 | NCT03294486 | Safety and Efficacy of the ONCOlytic VIRus Armed for Local Chemotherapy, TG6002/5-FC, in Recurrent Glioblastoma Patients | Phase 1/Phase 2 | Unknown | 78 | France | 2017/10/12-2021/9 | Drug: Combination of TG6002 and 5-flucytosine (5-FC, Ancotil®) | This is a Phase 1, open-label, dose-escalation trial using an accelerated titration 3+3 design in patients with recurrent glioblastoma to evaluate the safety of the combination of TG6002 and 5-flucytosine |
| 4 | NCT03714334 | DNX-2440 Oncolytic Adenovirus for Recurrent Glioblastoma | Phase 1 | Recruiting | 24 | Spain | 2018/10/16-2022/10/16 | Drug: DNX-2440 injection | Patients with first or second recurrence of GBM will be treated with stereotactic injection of the oncolytic virus DNX-2440. |
| 5 | NCT03896568 | Oncolytic Adenovirus DNX-2401 in Treating Patients With Recurrent High-Grade Glioma | Phase 1 | Recruiting | 36 | USA | 2019/2/12-2023/9/30 | Biological: Oncolytic Adenovirus Ad5-DNX-2401/Procedure: Therapeutic Conventional Surgery | This phase I trial studies best dose and side effects of oncolytic adenovirus DNX-2401 in treating patients with high-grade glioma that has come back (recurrent) |
| 6 | NCT02986178 | PVSRIPO in Recurrent Malignant Glioma | Phase 2 | Active, not recruiting | 122 | USA | 2017/6/1-2023/12 | Biological: PVSRIPO | This is a phase 2 study of oncolytic polio/rhinovirus recombinant (PVSRIPO) in adult patients with recurrent World Health Organization (WHO) grade IV malignant glioma. |
| 7 | NCT00028158 | Safety and Effectiveness Study of G207, a Tumor-Killing Virus, in Patients With Recurrent Brain Cancer | Phase 1/Phase 2 | Completed | 65 | USA | 2001/12-2003/10 | Drug: G207 | This clinical trial will study the safety and effectiveness of an engineered herpes virus, G207, administered directly into the brain of patients with recurrent brain cancer |
| 8 | NCT02197169 | DNX-2401 With Interferon Gamma (IFN-γ) for Recurrent Glioblastoma or Gliosarcoma Brain Tumors | Phase 1 | Completed | 37 | USA | 2014/9/11-2018/3/15 | Drug: Single intratumoral injection of DNX-2401/Drug: Interferon-gamma | The purpose of this Phase Ib study is to evaluate how well a recurrent glioblastoma or gliosarcoma tumor responds to one injection of DNX-2401, a genetically modified, conditionally replicative and oncolytic human-derived adenovirus. |
| 9 | NCT00528684 | Safety and Efficacy Study of REOLYSIN® in the Treatment of Recurrent Malignant Gliomas | Phase 1 | Completed | 18 | USA | 2006/7-2010/6 | Biological: REOLYSIN® | This phase I/II study investigates the maximum tolerated dose (MTD), dose limiting toxicity (DLT) and anti-tumor effect of intralesional administration of REOLYSIN® therapeutic reovirus in patients with malignant glioma with evaluable disease which is progressive/recurrent despite surgery and/or radiotherapy with or without chemotherapy. |
| 10 | NCT01491893 | PVSRIPO for Recurrent Glioblastoma (GBM) | Phase 1 | Completed | 61 | USA | 2012/4/25-2021/10/1 | Biological: Recombinant nonpathogenic polio-rhinovirus chimera (PVSRIPO) | To determine the maximally tolerated dose (MTD) and the Recommended Phase 2 Dose (RP2D) of PVSRIPO when delivered intracerebrally by convection-enhanced delivery (CED). |
| 11 | NCT03152318 | A Study of the Treatment of Recurrent Malignant Glioma With rQNestin34.5v.2 | Phase 1 | Active, not recruiting | 51 | USA | 2017/7/18-2023/12 | Drug: rQNestin/Drug: Cyclophosphamide/Procedure: Stereotactic biopsy | This research study is evaluating an investigational drug, an oncolytic virus called rQNestin34.5v.2. This research study is a Phase I clinical trial, which tests the safety of an investigational drug and also tries to define the appropriate dose of the investigational drug as a possible treatment for this diagnosis of recurrent or progressive brain tumor. |
| 12 | NCT01956734 | Virus DNX2401 and Temozolomide in Recurrent Glioblastoma | Phase 1 | Completed | 31 | Spain | 2013/9-2017/3 | Procedure: DNX2401 and Temozolomide | Phase I trial, unicentric, uncontrolled. Intratumoral injection or intramural (into the resected tumor cavity) of DNX2401 into brain tissue will be followed by up to two 28 - day cycles of oral temozolomide (TMZ) in schedule of 7 days on/7 days off to evaluate safety of the combination. |
| 13 | NCT05139056 | Multiple Doses of Neural Stem Cell Virotherapy (NSC-CRAd-S-pk7) for the Treatment of Recurrent High-Grade Gliomas | Phase 1 | Not yet recruiting | 30 | USA | 2022/7/15-2023/12/20 | Biological: Neural Stem Cells-expressing CRAd-S-pk7/Procedure: Resection | This phase I trial studies the effect of multiple doses of NSC-CRAd-S-pk7 in treating patients with high-grade gliomas that have come back (recurrent). |
| 14 | NCT05095441 | A Clinical Study of Intratumoral MVR-C5252 (C5252) in Patients With Recurrent or Progressive Glioblastoma | Phase 1 | Recruiting | 51 | USA | 2022/2/28-2026/4/30 | Biological: C5252 | This is a Phase 1 open label, first in human study of C5252 monotherapy designed to determine the safety and tolerability of a single intratumoral (IT) injection of C5252 in patients with recurrent or progressive glioblastoma (GBM). |
| 15 | NCT05084430 | Study of Pembrolizumab and M032 (NSC 733972) | Phase 1/Phase 2 | Active, not recruiting | 28 | USA | 2022/2/25-2025/3/1 | Drug: M032/Drug: Pembrolizumab | This Phase I (Cohort I and Cohort II) and Phase II trial is designed to confirm the safety and tolerability of Pembrolizumab when given in conjunction with M032, an Oncolytic Herpes Simplex Virus (oHSV) that expresses IL-12 and perform the Phase II portion using a Recommended Phase 2 Dose (RP2D) of M032 (provided by the Phase I) when given in conjunction with Pembrolizumab for recurrent malignant glioma (glioblastoma multiforme, anaplastic astrocytoma, or glio-sarcoma). |
| 16 | NCT02798406 | Combination Adenovirus + Pembrolizumab to Trigger Immune Virus Effects | Phase 2 | Completed | 49 | USA | 2016/10/6-2021/6/30 | Biological: DNX-2401/Biological: pembrolizumab | The purpose of this Phase II study is to evaluate how well a recurrent glioblastoma or gliosarcoma tumor responds to one injection of DNX-2401, a genetically modified oncolytic adenovirus, when delivered directly into the tumor followed by the administration of intravenous pembrolizumab (an immune checkpoint inhibitor) given every 3 weeks for up to 2 years or until disease progression. |
| 17 | NCT01582516 | Safety Study of Replication-competent Adenovirus (Delta-24-rgd) in Patients With Recurrent Glioblastoma | Phase 1/Phase 2 | Completed | 20 | Netherlands | 2010/6-2014/12 | Biological: delta-24-RGD adenovirus | The primary objective is to determine the safety and tolerability of Delta-24-RGD administered by CED to the tumor and the surrounding infiltrated brain in patients with recurrent GBM. |
| 18 | NCT03657576 | Trial of C134 in Patients With Recurrent GBM | Phase 1 | Recruiting | 24 | USA | 2019/9/23-2024/9 | Biological: C134 | The purpose of this project is to obtain safety information in small groups of individuals, scheduled to receive escalating doses of C134, a cancer killing virus (HSV-1) that has been genetically engineered to safely replicate and kill glioma tumor cells |
| 19 | NCT01301430 | Parvovirus H-1 (ParvOryx) in Patients With Progressive Primary or Recurrent Glioblastoma Multiforme. | Phase 1/Phase 2 | Completed | 18 | Germany | 2011/9-2015/5 | Drug: H-1PV | Investigation on safety, tolerability and efficacy of H-1 parvovirus (H-1PV) in subjects suffering from glioblastoma multiforme. |
| 20 | NCT02062827 | Genetically Engineered HSV-1 Phase 1 Study for the Treatment of Recurrent Malignant Glioma | Phase 1 | Active, not recruiting | 24 | USA | 2013/11/25-2023/9 | Biological: M032 (NSC 733972) | To determine the safety and tolerability of the maximum dose for laboratory engineered Herpes Simplex Virus-1 in patients who would not be eligible for surgical resection of recurrent glioma To determine the safety and tolerability of the maximum dose for laboratory engineered Herpes Simples Virus-1 in patients who would benefit from surgical resection of recurrent glioma |
| 21 | NCT00157703 | G207 Followed by Radiation Therapy in Malignant Glioma | Phase 1 | Completed | 9 | USA | 2005/5-2008/12 | Drug: G207 | This is an open-label, single site study to evaluate the safety and tolerability of intratumoral administration of G207 followed by treatment with radiation therapy in patients with recurrent/progressive malignant glioma. |
| 22 | NCT00805376 | DNX-2401 (Formerly Known as Delta-24-RGD-4C) for Recurrent Malignant Gliomas | Phase 1 | Completed | 37 | USA | 2009/2-2015/2 | Drug: DNX-2401/Procedure: Tumor Removal | The goal of this clinical research study is to find the highest tolerable dose of DNX-2401 that can be injected directly into brain tumors and into the surrounding brain tissue where tumor cells can multiply. |
| 23 | NCT00390299 | Viral Therapy in Treating Patients With Recurrent Glioblastoma Multiforme | Phase 1 | Completed | 23 | USA | 2006/10/23-2019/11/30 | Biological: Carcinoembryonic Antigen-Expressing Measles Virus/Procedure: Therapeutic Conventional Surgery | This phase I trial studies the side effects and best dose of carcinoembryonic antigen-expressing measles virus (MV-CEA) in treating patients with glioblastoma multiforme that has come back. |

**Supplementary Table 2: Pediatric clinical studies related to OVT-CNSTs**

| Study | NCT | Office title | Phase | Status | Number Enrolled | Country | Time | Interventions | Summary |
| --- | --- | --- | --- | --- | --- | --- | --- | --- | --- |
| 1 | NCT03043391 | Phase 1b Study PVSRIPO for Recurrent Malignant Glioma in Children | Phase 1 | Active, not recruiting | 12 | USA | 2017/12/5-2022/3/1 | Biological: Polio/Rhinovirus Recombinant (PVSRIPO) | The purpose of the study is to confirm the safety of the selected dose and potential toxicity of oncolytic poliovirus (PV) immunotherapy with PVSRIPO for pediatric patients with recurrent WHO grade III or IV malignant glioma, but evidence for efficacy will also be sought. |
| 2 | NCT03911388 | HSV G207 in Children With Recurrent or Refractory Cerebellar Brain Tumors | Phase 1 | Recruiting | 15 | USA | 2019/9/12-2025/9/1 | Biological: G207 | This study is a clinical trial to determine the safety of inoculating G207 (an experimental virus therapy) into a recurrent or refractory cerebellar brain tumor. |
| 3 | NCT02457845 | HSV G207 Alone or With a Single Radiation Dose in Children With Progressive or Recurrent Supratentorial Brain Tumors | Phase 1 | Active, not recruiting | 12 | USA | 2016/5-2022/12 | Biological: G207 | This study is a phase I, open-label, single institution clinical trial of G207 alone or combined with a single low dose of radiation in children with recurrent or progressive supratentorial brain tumors. |
| 4 | NCT03178032 | Oncolytic Adenovirus, DNX-2401, for Naive Diffuse Intrinsic Pontine Gliomas | Phase 1 | Unknown | 12 | Spain | 2017/5/26-2021/1/31 | Biological: DNX-2401 | This study is a phase I, unicentric, non-randomized clinical trial to study the safety and potential efficacy of intratumoral administration of DNX-2401 in DIPG. |
| 5 | NCT04758533 | Clinical Trial to Assess the Safety and Efficacy of AloCELYVIR With Newly Diagnosed Diffuse Intrinsic Pontine Glioma (DIPG) in Combination With Radiotherapy or Medulloblastoma in Monotherapy | Phase 1/Phase 2 | Recruiting | 12 | Spain | 2021/4/19-2024/10 | Biological: AloCELYVIR | The aim of this study is to assess the safety and efficacy of AloCELYVIR, which consist in bone marrow-derived allogenic mesenchymal stem cells infected with an oncolytic Adenovirus, ICOVIR-5. |
| 6 | NCT04482933 | HSV G207 With a Single Radiation Dose in Children With Recurrent High-Grade Glioma | Phase 2 | Not yet recruiting | 30 | USA | 2022/12/1-2026/12/1 | Drug: Biological G207 | This study is a phase II, open-label, single arm clinical trial of G207 alone or combined with a single low dose of radiation in children with recurrent or progressive high grade glioma. |
| 7 | NCT02031965 | Oncolytic HSV-1716 in Treating Younger Patients With Refractory or Recurrent High Grade Glioma That Can Be Removed By Surgery | Phase 1 | Terminated | 2 | USA | 2013/12-2016/5 | Biological: oncolytic HSV-1716/Drug: dexamethasone/Procedure: therapeutic conventional surgery | This phase I trial studies the side effects and the safety of injecting HSV1716 (a new experimental therapy) into or near the tumor resection cavity |
| 8 | NCT02444546 | Wild-Type Reovirus in Combination With Sargramostim in Treating Younger Patients With High-Grade Relapsed or Refractory Brain Tumors | Phase 1 | Active, not recruiting | 6 | USA | 2015/6/21-2025/1/1 | Biological: Sargramostim/Biological: Wild-type Reovirus | This phase I trial studies the side effects and the best dose of wild-type reovirus (viral therapy) when given with sargramostim in treating younger patients with high grade brain tumors that have come back or that have not responded to standard therapy |
| 9 | NCT02962167 | Modified Measles Virus (MV-NIS) for Children and Young Adults With Recurrent Medulloblastoma or Recurrent ATRT | Phase 1 | Recruiting | 46 | USA | 2017/2/22-2024/5/1 | Biological: Modified Measles Virus/Biological: Modified Measles Virus Lumbar Puncture | This is an open label, multi-center, Phase I study to assess the safety of administering MV-NIS directly into the tumor bed (for locally recurrent medulloblastoma or ATRT patients) or into the subarachnoid space (for disseminated recurrent medulloblastoma or ATRT patients). |
